# Transcriptomic and proteomic profiling of two porcine tissues using high-throughput technologies

**DOI:** 10.1186/1471-2164-10-30

**Published:** 2009-01-19

**Authors:** Henrik Hornshøj, Emøke Bendixen, Lene N Conley, Pernille K Andersen, Jakob Hedegaard, Frank Panitz, Christian Bendixen

**Affiliations:** 1 Department of Genetics and Biotechnology, Faculty of Agricultural Sciences, Aarhus University, DK-8830 Tjele, Denmark; 2 Department of Animal Health, Welfare and Nutrition, Faculty of Agricultural Sciences, Aarhus University, DK-8830 Tjele, Denmark

## Abstract

**Background:**

The recent development within high-throughput technologies for expression profiling has allowed for parallel analysis of transcriptomes and proteomes in biological systems such as comparative analysis of transcript and protein levels of tissue regulated genes. Until now, such studies of have only included microarray or short length sequence tags for transcript profiling. Furthermore, most comparisons of transcript and protein levels have been based on absolute expression values from within the same tissue and not relative expression values based on tissue ratios.

**Results:**

Presented here is a novel study of two porcine tissues based on integrative analysis of data from expression profiling of identical samples using cDNA microarray, 454-sequencing and iTRAQ-based proteomics. Sequence homology identified 2.541 unique transcripts that are detectable by both microarray hybridizations and 454-sequencing of 1.2 million cDNA tags. Both transcript-based technologies showed high reproducibility between sample replicates of the same tissue, but the correlation across these two technologies was modest. Thousands of genes being differentially expressed were identified with microarray. Out of the 306 differentially expressed genes, identified by 454-sequencing, 198 (65%) were also found by microarray. The relationship between the regulation of transcript and protein levels was analyzed by integrating iTRAQ-based proteomics data. Protein expression ratios were determined for 354 genes, of which 148 could be mapped to both microarray and 454-sequencing data. A comparison of the expression ratios from the three technologies revealed that differences in transcript and protein levels across heart and muscle tissues are positively correlated.

**Conclusion:**

We show that the reproducibility within cDNA microarray and 454-sequencing is high, but that the agreement across these two technologies is modest. We demonstrate that the regulation of transcript and protein levels across identical tissue samples is positively correlated when the tissue expression ratios are used for comparison. The results presented are of interest in systems biology research in terms of integration and analysis of high-throughput expression data from mammalian tissues.

## Background

High-throughput quantitative profiling of transcripts and proteins is a widely used approach for studying biological processes. As a consequence, technologies develop rapidly in order to improve quality, increase the throughput and reduce the cost of expression profiling. Currently, transcript profiling technologies include DNA microarray [1], Serial Analysis of Gene Expression (SAGE) [2], Massive Parallel Signature Sequencing (MPSS) [3] and recently the sequencing technologies from 454 Life Sciences (now Roche) [4] and Solexa (now Illumina). Hybridization-based microarray technologies have been the dominating method for transcript profiling and are characterized by their ability to globally profile gene expression in large numbers of tissue samples. We recently developed and applied the cDNA microarray technology in porcine studies of gene expression in multiple samples of diseased [5] and healthy [6] tissues. Microarray expression profiles are extracted from signal intensities reflecting the amount of hybridized mRNA to spotted DNA whereas the above mentioned sequencing-based technologies provide expression levels that are absolute values computed as the number of transcripts observed for individual genes. At the protein level, introduction of the iTRAQ-tagging approach, has allowed simultaneous quantitative comparison of individual protein levels in multiple tissue samples [7]. Currently the iTRAQ-based proteomics technology is not able to fully characterize entire proteomes [8], which is a limiting factor in global comparative studies of transcript and protein expression. Comparative analysis of protein expression in pig tissues using iTRAQ-based tagging was recently reported [9]. In comparison to SAGE, MPSS and Solexa, 454-sequencing has increased the sequence length to a minimum of 110 bp. The ability for transcript profiling across multiple tissue samples has been reported for most high-throughput sequencing-based technologies, but has been limited to single tissue profiling for 454-sequencing [10,11].

As new high-throughput technologies emerge and develop, more comparative expression studies across technologies and across transcriptomes and proteomes have been reported. At the transcript level, these studies have been dominated by comparisons of SAGE data with either Affymetrix short oligonucleotide microarrays [12-22] or cDNA microarrays [13,14,16,21,23,24]. A few studies have compared long oligonucleotide microarrays with SAGE [25,26] and MPSS [25,27]. In one study, several commercial oligonucleotide-based platforms were compared with MPSS [28]. As demonstrated by many previous studies, computation and evaluation of Pearson's or Spearman correlation coefficients allows for comparison of transcript-based expression profiles across technologies [13,15,17-22,25,28]. Comparison of transcript and protein profiling has been used in studies of various mammalian tissues including high productivity Chinese Hamster Ovary (CHO) cells [29], murine stem cell populations [30] and recombinant NS0 cells [31]. Studies in yeast have also been reported that integrate transcript and protein expression [32-35]. Integrative studies of transcriptomic and proteomic profiles by means of 454-sequencing and iTRAQ-based proteomics have not been reported. The reported levels of correlation in gene expression across technologies have been fairly inconsistent. The observed discrepancies between transcript-based technologies have been suggested to result from errors in SAGE tag-to-gene mapping, errors in microarray probe-to-gene mapping [19,22] and alternative polyadenylation [17]. The correlation between quantitative proteomics data and microarray data may be affected by alternative splicing [8]. Studies of the correlation between the regulation of transcriptomes and proteomes have mostly used direct comparisons of absolute measurements within single tissues where the differences across genes in translational efficiencies, turn-over rates and half life will have great impact on the level of agreement. On the other hand, it seems appropriate to assume that, when comparing the transcript and protein level of a gene in two tissues, the tissue with the highest transcript level will also be the tissue with the highest protein level. Hence, tissue expression ratios should be more compatible measurements when analyzing the relationship between transcript and protein abundances.

Here we report a comparative study of three high-throughput technologies for multi-sample expression profiling using tissue samples from pig heart and skeletal muscle. We compare transcript profiles from 454-sequencing and cDNA microarray based on relative expression within tissues and expression ratios across tissues. Furthermore, we analyze the relationship between transcript and protein regulation between the two tissues by direct comparison of expression ratios from cDNA microarray, 454-sequencing and iTRAQ-based proteomics.

## Results

### Expression profiles for genes overlapping between technologies

**Figure 1:**
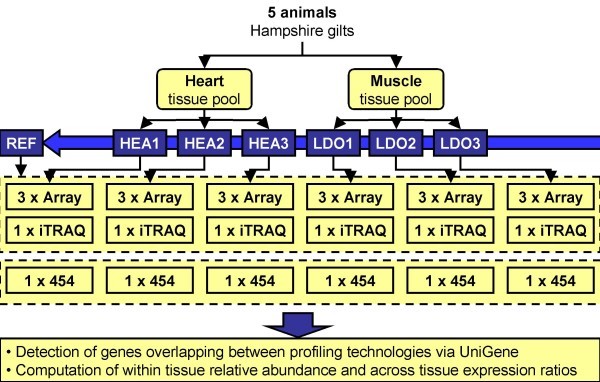
**Overview of the experimental design**. For microarray and iTRAQ, a common reference sample (REF) was constructed by combining the three samples from heart (HEA1, HEA2 and HEA3) and three samples from the skeletal muscle *Longissimus dorsi *(LDO1, LDO2, and LDO3). In the microarray experiment, three cDNA microarray slides were used per sample.

Identical tissue samples were used in expression profiling with 454-sequencing, cDNA microarray and iTRAQ-based proteomics, three for heart and three for skeletal muscle. An overview of the experimental setup is shown in figure 1. For cDNA microarray and iTRAQ-based proteomics, a common reference sample was constructed by mixing equal aliquots from all six samples facilitating computation of relative expression profiles and expression ratios across tissues. For 454-sequencing data, relative expression profiles were computed from absolute transcript count per UniGene ID identified by sequence homology. Expression profiles were compared across technologies by matching unique pig UniGene sequence IDs as the gene IDs from the different expression sets. The UniGene IDs for 454-sequencing and microarray cDNAs were identified by BlastN whereas the UniGene IDs for the iTRAQ-based proteins were identified by searching mass spectrometry data against the database of predicted proteins from UniGene sequences downloaded at trEST [36]. Table 1 shows the number of UniGene IDs identified by microarray cDNA, 454-sequencing and the overlapping UniGene IDs between the two transcript-based technologies. A major portion of the UniGene IDs identified by 454-sequencing is overlapping with UniGene IDs represented by microarray cDNAs. Of 26.877 cDNA sequences, we were able to map 12.563 cDNAs to unique UniGene IDs. A total of 1.253.361 sequences were generated using the 454-based technology divided into 551.666 for heart and 701.695 for skeletal muscle. In total, 647.093 sequences were mapped to 18.624 UniGene IDs. The expression profiles from 454-sequencing were obtained by BlastN sequence homology between 454 sequence reads and UniGene database sequences. Absolute gene expression was determined by counting the number of transcripts per gene, in this case computed as the number of 454 sequences for each UniGene ID in each of the six samples. In total, 2.954 UniGene IDs were found to have sequence counts in all six samples. We were able to compare expression profiles from microarray and 454-sequencing for 2.541 UniGene IDs by using the matching IDs. Relative expression profiles for 454-sequencing were represented by Relative Abundance (RA) values computed as the absolute sample sequence count divided by the total sequence count in that sample. Microarray RA values were computed as the sample signal intensity divided by the total sample signal intensity. RA values have previously been used as estimates of relative gene expression for rough comparisons within and across expression platforms [6,37]. Logarithmic transformed RA values were used for subsequent analysis of differential gene expression using 454-sequencing. With the iTRAQ-based proteomics we identified proteins corresponding to 354 UniGene IDs. In terms of matches between protein identifications from iTRAQ-based proteomics and transcript profiles, we were able to map 148 UniGene IDs to 454-sequencing, 202 UniGene IDs to microarray cDNAs and 148 UniGene IDs to both 454-sequences and microarray cDNAs.

**Table 1:** Summary of 454-sequencing and overlap to microarray cDNAs via pig UniGene sequence IDs

**RNA** **Sample**	**454 sequence** **count**	**454 sequence** **count with** **UniGene hit**	**Unique UniGene** **sequence count**	**Unique UniGene sequence count with array overlap**
HEA1	173.571	79.401	10.567	6.544
HEA2	91.591	37.944	6.551	4.873
HEA3	286.504	134.455	12.783	7.426
Subtotal	551.666	251.800	15.885	8.764
				
LDO1	258.307	142.498	10.116	6.290
LDO2	155.443	85.158	7.555	5.098
LDO3	287.945	167.637	9.320	6.384
Subtotal	701.695	395.293	14.059	8.230
				
Total	1.253.361	647.093	18.624	9.741
Total, N ≥ 1		471.028	2.954	2.541

N ≥ 1 indicates that these UniGene IDs are identified by BlastN at least once in all six RNA samples by 454-sequencing.

### Reproducibility of gene expression within and across 454-sequencing and cDNA microarray

**Figure 2:**
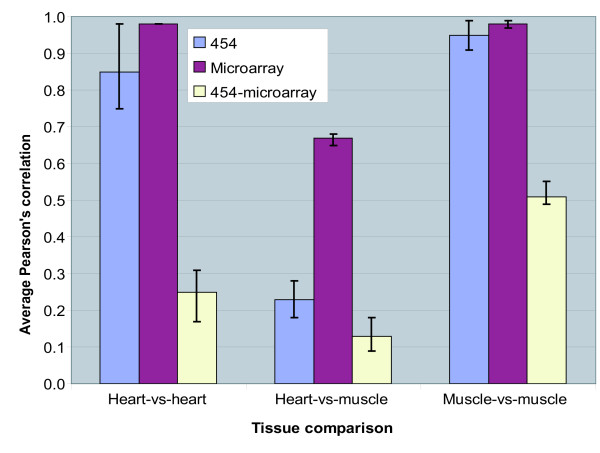
**Reproducibility within and across transcript-based technologies**. Average correlation between transcript RA profiles from heart and skeletal muscle tissues within and across the two transcript-based technologies 454-sequencing and cDNA microarray. Black vertical error bars indicate the minimum and maximum values of the Pearson's correlations used to calculate average correlation.

To determine the level of reproducibility of 454-sequencing and cDNA microarray for measuring gene expression in heart and skeletal muscle and to evaluate the level of correlation in expression across the two technologies, Pearson's correlations between RA for pairs of tissue samples for the 2.541 shared genes were computed. For each of the six samples and two technologies, a vector of RA expression values was prepared. The resulting 12 RA expression vectors were then used to compute pair-wise tissue sample correlations, of which the average values within and across the two tissues and technologies are plotted in figure 2. Both technologies show good reproducibility within the same tissue shown by the high correlation values. For measuring expression in heart the average correlation is 0.85 with 454-sequencing and 0.98 with cDNA microarray. The corresponding values for skeletal muscle were found to be 0.95 for 454-sequencing and 0.98 for cDNA microarray. Although high reproducibility was observed with both technologies, microarray has a slightly higher reproducibility than 454-sequencing for measuring these two particular tissues. The correlation within cDNA microarray is relatively high across the two tissues with average correlation at 0.67 in comparison to the average correlation at 0.23 observed for 454-sequencing. To further analyze this difference between 454-sequencing and microarray, a comparison of the distribution of RA values was made. This revealed that 454-sequencing generates expression profiles with higher dynamic range in comparison to microarray, indicating that the relative expression profiles measured by 454-sequencing are more different (Additional file 1). Across 454-sequencing and microarray, the highest correlation in gene expression was observed between skeletal muscle samples with an average value at 0.51. The correlation across 454-sequencing and microarray for measuring the gene expression in heart was less pronounced with an average value at 0.25. These correlations are relatively low, but are still higher than the average correlation value at 0.13 across heart and muscle using the two different transcript-based technologies as would be expected.

### Level of agreement between 454-sequencing and cDNA microarray in detecting differentially expressed genes

**Figure 3:**
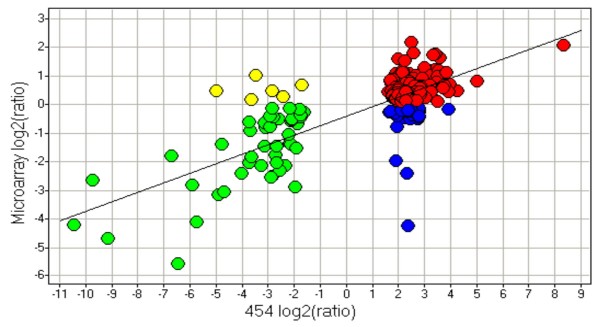
**Comparison of differentially expressed genes across transcript-based technologies**. Level of agreement between 454-sequencing and cDNA microarray in the direction of regulation for 198 genes identified as significantly (adj. P ≤ 0.05) regulated between heart and skeletal muscle by both technologies. The scatter plot shows the log2 of the ratios between heart and skeletal muscle obtained with 454-sequencing plotted against those obtained with cDNA microarray. Red spots: 113 genes being up-regulated in heart compared to skeletal muscle were identified with both 454-sequencing and cDNA microarray. Green spots: 47 genes being down-regulated in heart compared to skeletal muscle were identified with both 454-sequencing and cDNA microarray. Yellow spots: 6 genes being up-regulated in heart compared to skeletal muscle were identified with cDNA microarray but found to be down-regulated with 454-sequencing. Blue spots: 32 genes being up-regulated in heart compared to skeletal muscle were identified with 454 but found to be down-regulated with cDNA microarray.

The agreement between 454-sequencing and cDNA microarray in identifying differential gene expression was evaluated by comparing lists of differentially expressed genes between heart and skeletal muscle. A t-test was carried out to compute the fold changes and P-values corrected for multiple testing was set to 0.05 as a cutoff for significant regulation. Identification of differentially expressed genes using 454-sequencing produces the lowest number of significantly regulated genes, 306 out of 2.954 tested genes (10%). Out of the tested 12.563 genes, microarray produces a list of 5.888 genes that are significantly regulated, a much higher percentage (47%) compared to that of 454-sequencing. Of the 306 significantly regulated genes identified using 454-sequencing, 198 genes (65%) are overlapping with the 5.888 genes identified using cDNA microarray. Plotting the log2 of the expression ratios between heart and skeletal muscle for these 198 genes shows that the direction of the regulation is identical for a majority of the genes (figure 3). Out of 198 genes, 160 (81%) are regulated in the same direction using both 454-sequencing and cDNA microarray. However, some discrepancy was found between these two technologies in terms of the direction of the regulation. For instance, 6 genes were found to be up-regulated in heart compared to skeletal muscle with microarray, whereas 454-sequencing identified these genes as being down-regulated. Similarly, 32 genes were found to be up-regulated in heart compared to skeletal muscle according to 454-sequencing but found to be down-regulated with microarray. Thus, out of 198 genes, 38 (19%) genes were significantly regulated in both technologies but in the opposite direction. The plot in figure 3 also demonstrates a higher dynamic range of the expression ratios for 454-sequencing than for cDNA microarray.

### Correlation between heart-muscle expression ratios across transcript-based technologies and iTRAQ-based proteomics

**Figure 4:**
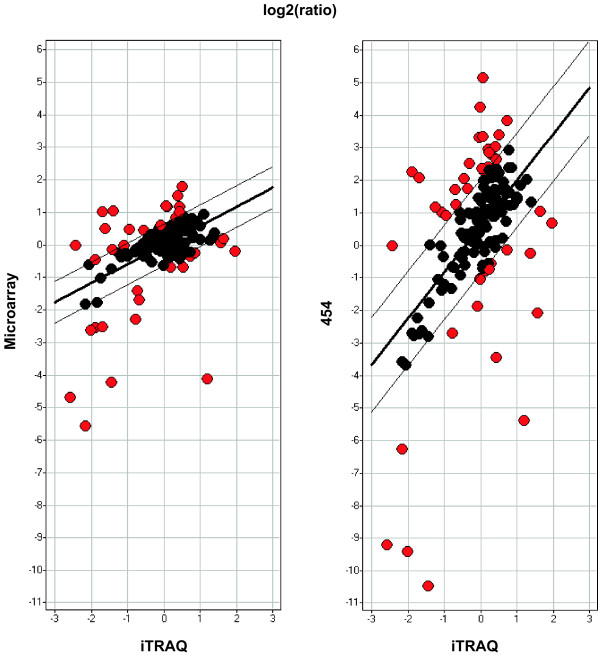
**Comparison of tissue expression ratios from transcriptomic and proteomic profiling**. Pair-wise comparison of heart-muscle log2 expression ratios obtained by 454-sequencing, cDNA microarray and iTRAQ-based proteomics for 148 genes detectable by all three technologies. Thick lines represent fitted straight lines from the data points. The distances between the thick and thin lines are equal to one time the standard deviation of all distances from data points to the fitted lines. The black data points between the two thin lines were considered to represent genes where transcript and protein ratios are in concordance, whereas the outside red data points were considered to represent genes where transcript and protein ratios are not in concordance (red).

To investigate the level of agreement between transcript and protein ratios, heart-muscle expression ratios were extracted for the two transcript-based technologies and pair-wise comparisons were made to protein ratios obtained from iTRAQ-based proteomics. Using the UniGene IDs it was possible to link expression ratios for 148 genes across the three technologies. The pair-wise ratio comparisons for cDNA microarray versus iTRAQ and 454-sequencing versus iTRAQ with indications of transcript and protein concordance are shown in figure 4. The slopes for the fitted straight lines for cDNA microarray and 454-sequencing were calculated to be 0.6 and 1.4 respectively. The corresponding Pearson's correlation coefficients were positive and computed to be 0.49 and 0.53 respectively, indicating that 454-based mRNA expression ratios are slightly more in agreement with the protein ratios in comparison to mRNA expression ratios produced by the cDNA microarray platform. We made a list of the 148 overlapping genes and classified each gene in terms of concordance between transcript and protein ratios based on the distance of the data point to the fitted straight line and a cutoff value at one time the standard deviation of all distances (Additional file 2). In the comparison of iTRAQ and 454-sequencing, 43 UniGene IDs are not in concordance and the corresponding number for the comparison of iTRAQ and microarray was 34 UniGene IDs. The observed positive correlations between transcript and protein ratios for both transcript-based technologies demonstrate that for most genes differences in transcript levels across heart and skeletal muscle is accompanied by similar differences in the protein level.

## Discussion

In this study, cDNA microarray and 454-sequencing have been compared for transcript expression profiling in porcine heart and skeletal muscle tissues. Protein abundance data for identical tissue samples was generated using iTRAQ-based proteomics to analyze the relationship within genes between transcript and protein ratios across tissues.

A total of 2.541 genes were detectable by both transcript-based technologies. The degree of overlapping genes detectable by both cDNA microarray and 454-sequencing is affected by the nature of the two technologies. With cDNA microarray, spot intensities are provided for all genes printed on the microarray slides, regardless of whether the genes are expressed or not. In case of 454-sequencing, expression of a gene can be measured if the gene is transcribed and if the sequencing depth of the experiment is sufficiently high. For example, low expressed genes will require that more sequences are generated in order to be captured. Thus, the degree of overlap will depend largely on the genes being expressed in the particular tissues being examined. In order to compare t-tests for differential expression with cDNA microarray we restricted the 454-sequencing data set to genes with expression values in all six tissue samples, which reduced the number of genes and thereby the number of overlapping genes. This restriction may have removed informative data, but was necessary to allow comparison.

The reproducibility in profiling gene expression across replicates of the same tissue sample is high for both transcript-based technologies with correlation values at 0.85 for 454-sequencing and 0.98 for cDNA microarray. The expression correlation between heart and skeletal muscle within cDNA microarray is relatively high (0.67) in comparison to the corresponding correlation observed for 454-sequencing (0.23), which suggests a larger tissue difference. We speculate that the main reason for the difference between these two transcript-based technologies is the differences in the dynamic range of the expression profiles. Thus, the distribution of the RA values from 454-sequencing is considerably wider than that observed for microarray, indicating that sensitivity for detecting expression difference across tissue samples is highest for 454-sequencing. Whether the larger number of genes detected to be different across skeletal muscle and heart by 454-sequencing is reflecting real biological differences between these two tissues is uncertain. The correlation values across 454-sequencing and microarray at 0.25 and 0.51 for heart and muscle respectively was relatively low, but considerably higher than the correlation value at 0.13 across the two transcript-based technologies and across the two tissues. This means that even though the technologies are quite different they still measure the respective expression profiles within heart and muscle to be most similar.

The number of differentially expressed genes identified in relation to the number of tested genes was much higher for cDNA microarray (47%; 5.888 out of 12.563) than for 454-sequencing (10%; 306 out of 2.954). Because it is not the same set of genes being tested, it will of course not lead to the same list of identified differentially expressed genes. However, microarray tends to predict more genes that are differentially expressed. Some might be false positives, even though P-values were corrected for multiple testing. If expression ratios across any given two tissues are close to one, they are vulnerable to random shifts between up-regulated, not-regulated and down-regulated genes. This has previously been suggested as a reason for discrepancy between cDNA- and oligonucleotide-based microarray platforms in expression ratios [38]. Cardiac and skeletal muscles represent two different tissues, although they can both be classified as muscle type tissues. The fact that only about 10% of the monitored genes with 454-sequencing are differentially expressed may not be surprising due to the similarity of these two tissues. Thus, genes that are related to basic functions in muscle tissue cells would be expected to be expressed in both tissues. A relatively low number of differentially expressed genes between similar tissue samples has previously been observed in a study of rhabdomyosarcoma, fetal and skeletal muscle tissues, where 403 tags out of 41084 unique tags were identified as differentially expressed, although this study was based on the SAGE technology [39]. The fact that cDNA microarray has a lower dynamic range suggests that this technology is less sensitive to minor differences in gene expression across tissues than the sequencing-based approach. However, microarray detects more genes than 454-sequencing that are differentially expressed across cardiac and skeletal muscle. One explanation may be that if the variation in expression across sample replicates within tissue is considerably lower than the variation across tissues, significantly regulated genes can still be detected. This is supported by the high reproducibility observed for microarray within tissues in figure 2. Of the 306 genes identified as being differentially expressed with 454-sequencing, 198 was also identified by microarray and 160 were regulated in the same direction. Out of 198 genes found to be significantly regulated between the two tissues, 38 genes are regulated in opposite direction with the two transcript-based technologies. This may also be due to random shifts between positive and negative values in the log2 ratio in either of the two technologies. Other reasons for the disagreement in detection of differential expression could be gene mapping errors or alternative splicing events.

To investigate the relationship between the regulation of transcript and protein levels across tissues, the expression ratios across heart and skeletal muscle from microarray, 454-sequencing and iTRAQ-based proteomics were compared. Transcript and protein ratios across heart and skeletal muscle were shown to be positively correlated for 148 genes detectable by all three technologies. The correlation appear to be slightly more positive for the comparison with 454-sequencing (0.53) than for the comparison with cDNA microarray (0.49). The slope is 0.6 for cDNA microarray and 1.4 for 454-sequencing when ratios are compared against iTRAQ-based ratios. The positive correlation between transcript and protein ratios suggests that for most genes, a relative tissue difference across a given tissue pair in transcript level leads to a similar difference in the protein level. A list of all 148 genes with expression ratios for the three technologies and indications of concordance was generated. Genes, where the transcript-protein relationship deviates from this linearity might be interesting to study further, since they could be examples of differential regulation of either translation or mRNA- or protein-turnover among the tissues. Alternatively, translation of mRNAs from genes that generate high levels of transcripts might be limited by the availability of RNA-binding factor or the capacity of the translational machinery. Deviations may also result from gene annotation errors [19,22] or alternative splicing events taking place differentially across the two tissues, which are not equally detected by the transcript-based technologies and the iTRAQ-based technology [8]. Discrepancies arising from alternative splicing may potentially be verified with real time PCR. Future improvement of the proteomics-based technologies should also increase the depth of comparative studies of transcripts and proteins profiles. While the analysis described here allows for the detection of genes with alternative regulation in different tissues, further experiments are needed in order to understand the biological basis of these differences.

## Conclusion

In this study we have analyzed the reproducibility of expression data within and between microarray and 454-sequencing technologies. Furthermore, we have integrated expression data from both transcriptomic and proteomic profiling to analyze the gene regulation across two porcine tissues by comparing tissue expression ratios of transcripts and proteins. Both transcript-based technologies displayed a high degree of reproducibility within technology, but the reproducibility across these two technologies was modest. The majority of the differentially regulated genes identified by 454-sequencing was also found by the cDNA microarray platform. Most interesting was the comparison of data from both transcript-based technologies with relative expression values from iTRAQ-based proteomics. Integrative analysis revealed that the regulation of transcript and protein levels across the two tissues is positively correlated for most genes using tissue expression ratios for comparison. Some genes without transcript-protein concordance were identified, which may arise from annotation errors or differential regulation of translation, turnover or alternative splicing. The results presented here should be of high importance for integration and analysis of high-throughput expression data, in particular for studies of the regulation of transcript and protein abundances in mammalian tissues.

## Methods

### Tissue samples of heart and skeletal muscle

Tissue samples of heart (HEA) and skeletal muscle (*Longissimus dorsi*; LDO) were prepared by pooling equal amounts of tissue sampled from five healthy Hampshire gilts at age four to six months. Each pool was divided into six sub-samples, three for each tissue named HEA1, HEA2, HEA3, LDO1, LDO2 and LDO3. The exact same six tissue sub-samples were used for expression profiling with cDNA microarray, 454-sequencing and iTRAQ-based proteomics. A reference sample for the cDNA microarray experiment and iTRAQ-based proteomics was constructed by combining equal amounts of tissue from the six sub-samples.

### Microarray experiment

Total-RNA extractions were carried out from each sample using the RNeasy Maxi Kit from Qiagen. Alexa Flour-labelled cDNA was synthesized from 20 μg of total-RNA using SuperScript Plus Direct cDNA Labeling System from Invitrogen. The reference sample was labelled with Alexa 555 and the individual tissue samples were labelled with Alexa 647. Each of the six labeled tissue samples was co-hybridized with the labelled reference sample to three 27 k pig cDNA microarray slides representing approximately 20 k genes. Microarray cDNA platform development was based on a large EST sequence resource established as part of the Sino-Danish Pig Genome Sequencing Project [40,41]. Detailed description of the pig cDNA microarray platform can be found at NCBI's Gene Expression Omnibus (GEO; ) [42] using the accession ID GPL3585. Following hybridization, washing and drying, the slides were scanned and the output images were analyzed. Using the limma package [43] from Bioconductor , the log2-transformed median intensity ratios of Alexa-647 to Alexa-555 were normalized within-slide using the printtip-loess method [44]. The raw and normalized cDNA microarray gene expression data set was submitted to GEO at NCBI  and given the accession ID GSE10122.

### Preparation of cDNA libraries and 454-sequencing

From the same total-RNA extraction batches used for the microarray experiment, poly A+ RNA was purified from ~200 μg total-RNA using the Oligotex mRNA Mini Kit from Qiagen. The poly A+ RNA isolates were used as templates in synthesis of high quality double-stranded cDNA with oligo(dT)12–18-priming of the first strand cDNA (SuperScriptTM Doubled-Stranded cDNA Synthesis Kit from Invitrogen). The final purification of the synthesized cDNA was performed on MinElute PCR Purification column from Qiagen, and an average cDNA fragment size of 300–400 bp was obtained by applying 45 psi (= 3 bar) of nitrogen for 50–60 seconds on 2.7–4.7 μg starting material using a nebulizer. Subsequently, the distribution of fragments was profiled on a BioAnalyzer DNA 1000 LabChip (Agilent Technologies). As part of the library preparation, the ends of the sheared cDNA fragments were polished by treatment with T4 DNA Polymerase and T4 Polynucleotide Kinase prior to ligation of sequencing adaptors as previously described. The fragments were immobilized on streptavidin beads, following nick repair with Bst DNA Polymerase, and the sstDNA libraries were eluted by alkali denaturation. Finally, the qualities and quantities of the libraries were assayed on Bioanalyzer RNA 6000 Pico LabChip (Agilent Technologies). A titration run, based on DNA/bead ratios of 4 and 16 copies per bead (cpb) in the emulsion-based clonal amplification, revealed the optimal experimental set-up for the large sequencing run using enriched beads. Each of the six libraries was sequenced on a full 70 × 75 PicoTiterPlate (PTP). In total 1.253.361 sequences were generated and submitted to the Short Read Archive (SRA) of NCBI and assigned the accession ID SRA000267.

### iTRAQ-based proteomics

Eight samples (including two reference samples) were processed in parallel. Total protein fraction was purified from 200 mg tissues as previously described [9]. The supernatant was stored at 80°C until use. Tryptic digestion (100 μg protein from each sample) and tagging with iTRAQ reagents (Applied Biosystems, Forster City, CA, USA) was performed as previously described [9]. The two reference samples were labelled with mass-tag 114, and then pooled and split for normalization. Samples were combined in a 1:1:1:1 ratios into two parallel 4-plexed samples, with each 4-plex containing a common reference sample, as well as both heart and skeletal muscle samples. Inclusion of a common reference sample in every 4-plex sample allowed comparison of the protein expression ratios across the different 4-plexes. Fifty (50) μg of iTRAQ-labelled peptides were separated by 2D-HPLC (Agilent Technologies, Palo Alto, CA, USA) according to detailed descriptions [9]. The eluted peptides were sprayed through a nanospray needle (Fused Silica Emitters, OD 360 μm, ID 75 μm, Proxeon Biosystems, Odense, Denmark) directly into the Q-star XL mass spectrometer (Applied Biosystems, Forster City, CA, USA). The raw data files were searched with the Protein Pilot 1.0 software (Applied Biosystems) using the ParagonTM algorithm for protein grouping and confidence scoring, and searched against a database of proteins predicted from pig UniGene release 30 downloaded at trEST [36]. There was no processing (e.g. smoothing) of the raw data files prior to database searching. The database allowed for iTRAQ reagent labels at N-terminal residues, internal K and Y residues, and MMTS-labelled cysteine as fixed modifications, deamidation, O-phosphorylation (STY) and oxidation (M) as variable modifications and one missed cleavage. Confidence of protein identification was selected according to a 95% confidence and a minimum of two peptides identified per protein.

### Identification of IDs for genes detected across expression technologies

The microarray cDNA gene expression data and the 454-sequencing data was linked together by mapping all microarray cDNA sequences and 454-based sequences to NCBI's pig UniGene database release 28 and then linking the data by their shared UniGene IDs. The sequences were mapped to UniGene IDs by BlastN and collecting only IDs for the first hit with score at or above 100. For correct microarray cDNA mappings it was required for each UniGene Id that the corresponding UniGene sequence was able to identify the exact same microarray cDNA sequence as the first hit when it was compared back to a database of all microarray cDNA sequences. In total, 12.563 out of 26.877 microarray cDNA sequences were mapped to 12.563 UniGene IDs and 647.093 out of 1.253.361 454-sequences were mapped to 18.624 UniGene IDs. In total, 2.541 genes were identified with expression profiles from both cDNA microarray and 454-sequencing by using the overlapping UniGene IDs to merge the two data sets together. For iTRAQ-based proteomics, a database of predicted pig proteins from trEST based on translations of the latest release (30) of UniGene sequences was downloaded and searched [36]. Using this database, proteins corresponding to 356 UniGene IDs were identified and 148 of these could be directly linked to 454-sequencing, whereas 202 IDs could be linked to the microarray cDNAs. The total overlap between all three technologies consisted of 148 UniGene IDs.

### Computation of relative expression values for 454-sequencing and microarray

The expression profiles from 454-sequencing was achieved by counting the number of transcripts per gene, in this case represented by the number of BlastN query 454-sequences for each target UniGene ID in each of the six tissue samples. The minimum number of sequence counts in a tissue sample was set to one resulting in a total number of 2.954 UniGene IDs for which gene expression could be detected by 454-sequencing in all six samples. To calculate within-tissue relative expression profiles for each of the six tissue samples, taken into account the variation in total raw sequence counts, Relative Abundance (RA) values were computed for each gene (UniGene ID) in each tissue sample. The RA value for a given gene in a given tissue sample was computed as the 454-sequence count for that gene divided by the total 454-sequence count for the 2.541 genes in the whole tissue sample. To be able to make direct comparison of 454-based RA values with microarray-based expression profiles for the six tissue samples, corresponding microarray RA values were also computed using the same approach. The microarray RA value for a given gene was computed as previously proposed [6,37] based on the raw median signal intensity for Alexa 647 divided by the total signal intensity for Alexa 647 in that tissue sample. Three microarray slides were used per tissue sample so an average RA value was calculated for each tissue sample and used for comparison. Computation of RA values was based the 2.541 genes detected by both 454-sequencing and cDNA microarray.

### Computation of expression correlation values

For comparison of relative expression profiles across 454-sequencing and cDNA microarray we computed tissue sample correlations for examining the reproducibility within and across technologies. Twelve RA vectors, six RA vectors from 454-sequencing and the six RA vectors from cDNA microarray, corresponding to the six tissue samples were paired in 12 × 12 = 144 possible combinations and the Pearson's correlation coefficient between the vectors in each combination was computed. The correlation values were grouped into nine bins corresponding to the three tissue comparisons (heart versus heart, heart versus skeletal muscle and skeletal muscle versus skeletal muscle) and the three technology comparisons (within 454-sequencing, within cDNA microarray and across 454-sequencing/cDNA microarray). Average correlations were computed for these nine bins and used to evaluate the reproducibility within and across 454-sequencing and cDNA microarray. For comparing across-tissue mRNA expression ratios from the two transcript-based technologies against iTRAQ-based protein ratios we first prepared log2-transformed values of the expression ratios between heart and skeletal muscle for all three technologies. These values were organized in three vectors and pair-wise Pearson's correlation coefficients were calculated for the two comparisons 454-sequencing versus iTRAQ-based proteomics and cDNA microarray versus iTRAQ-based proteomics. In the comparison of expression ratios across transcripts and proteins a straight line was fitted for the data points described by the standard equation Ax+By+C = 0, where -A/B is the slope and C is the intersection between the line and the y-axis. Distances for all data points (m, n) to the fitted line were calculated based on the standard formula d = |Am+Bn+C|/√(A^2^+B^2^). These distances were used to evaluate the concordance of genes across transcript and protein ratios.

### Identification of differentially expressed genes

Statistical analysis was carried out in the R computing environment version 2.6.0 using the limma package [43] version 2.11.14 from Bioconductor. To identify differentially expressed genes between heart and skeletal muscle, normalized log2-transformed ratio between tissue and reference were used for cDNA microarray and log2-transformed RA values for 454-sequencing. The empirical Bayes method was applied and the P-values were adjusted for multiple testing using the false discovery rate ("fdr"). A 5% significance level (adjusted P-values ≤ 0.05) threshold was applied for differentially expressed genes.

## Authors' contributions

HH have made the main contributions to the data analysis, data interpretation and drafting of the manuscript. EB has contributed with preparation of the proteomics data and evaluation of the manuscript. LNC conducted the microarray experiment and data processing. PKA conducted the 454-sequencing experiment. JH contributed with data interpretation and manuscript evaluation. FP contributed to the evaluation of the manuscript. CB supervised the experimental design and contributed to the data interpretation and manuscript evaluation. All authors read and approved the final manuscript.

## Supplementary Material

Additional File 1 **Distribution of transcript relative abundance from microarray and 454-sequencing.** Image file showing the distribution of RA values from muscle (grey) and heart (white) generated using microarray and 454-sequencing. RA values were log2 transformed and binned for optimal visualization. Click here for file

Additional File 2 **List of 148 overlapping genes between microarray, 454-sequencing and iTRAQ-based proteomics with indication of concordance between transcript and protein expression ratios.** Table file with details for 148 genes overlapping between microarray, 454-sequencing and iTRAQ-based proteomics. Table contains number, UniGene ID, name, log2 ratios for the three technologies and indications of concordance between transcript and protein expression ratios. Concordance was based on the distance between a point and the fitted straight line being less than one time the standard deviation of all distances. The table is sorted so that the genes that are not in concordance according to both 454-sequencing and microarray are listed first. Click here for file
