# Supplementary figures and images for: Transcriptomic and proteomic profiling of two porcine tissues using high-throughput technologies
Source: BMC Genomics. 2009 Jan 19;10:30. doi: 10.1186/1471-2164-10-30 (PMC2633351; doi:10.1186/1471-2164-10-30)

## Slide 1
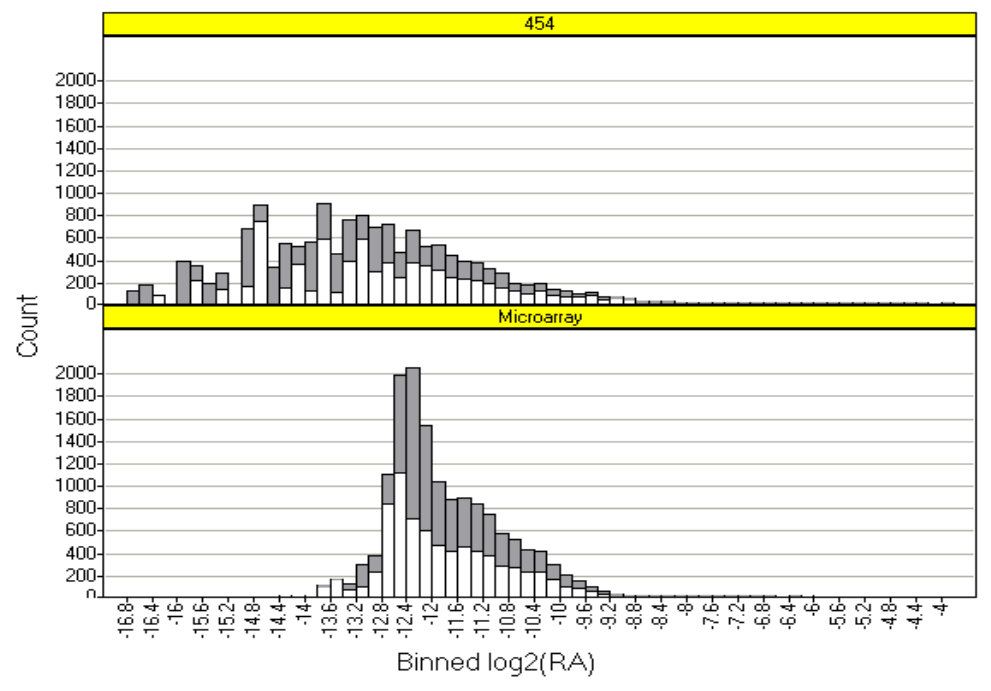

Supplement: Additional File 1 — Distribution of transcript relative abundance from microarray and 454-sequencing. Image file showing the distribution of RA values from muscle (grey) and heart (white) generated using microarray and 454-sequencing. RA values were log2 transformed and binned for optimal visualization. [file 1471-2164-10-30-S1.ppt]
